# General practice care following acute exacerbations of COPD: A survey of Australian general practitioners

**DOI:** 10.1371/journal.pone.0284731

**Published:** 2023-04-25

**Authors:** Bianca Perera, Chris Barton, Christian Osadnik

**Affiliations:** 1 Monash University, School of Primary and Allied Health Care, Frankston, VIC, Australia; 2 Monash University, School of Public Health and Preventive Medicine, Frankston, VIC, Australia; 3 Monash Lung, Sleep, Allergy, Immunology, Monash Health, Frankston, VIC, Australia; National and Kapodistrian University of Athens, GREECE

## Abstract

Acute exacerbations of COPD (AECOPDs) are one of the leading causes of preventable hospital admissions in Australia. Exacerbations are the strongest predictor for future exacerbations. The period immediately following an exacerbation is a high-risk period for recurrence and critical time to intervene. The aim of this study was to identify current general practice care for patients following an AECOPD in Australia and gain insights into knowledge of evidence-based care. A cross-sectional survey was created and disseminated electronically to Australian general practitioners (GPs). Data were analysed descriptively. Comparisons between groups were made using Chi squared tests. From 64 responses, 47% were familiar with the COPD-X Plan. Only 50% described reviewing patients within seven days of discharge mostly related to a lack of awareness of the hospital admission. 50% of surveyed GPs reported hospital discharge summaries did not provide the information they required. Smoking, immunisation and medications were regularly assessed by >90% respondents at follow-up visits, while referrals to pulmonary rehabilitation, and evaluation of spirometry and oxygen therapy were not prioritised. GPs appear to require support to increase their familiarity with COPD guidelines and inform evidence-based clinical practice. The handover/communication process from hospital to primary care appears an important area for future improvement.

## Introduction

Chronic obstructive pulmonary disease (COPD) is a major health problem in Australia and worldwide. About 1 in 20 Australians aged 45 years and over had COPD in 2017–18 [1]. Most importantly, COPD is one of the leading causes of preventable hospital admissions which are largely driven by severe acute exacerbations [2].

Acute exacerbations of COPD are common occurrences which involve sustained worsening of symptoms that are beyond normal day to day variations and necessitate change in regular medication [3–5]. Exacerbations become more frequent with worsening of COPD severity and are the strongest predictor for future exacerbations [6]. Re-exacerbations are commonly attributed to incomplete recovery. The period immediately following an exacerbation is therefore a critical time to intervene to disrupt this pattern [7,8]. Australia and New Zealand’s Guidelines for the management of COPD (The COPD-X guidelines) clearly describe the importance of comprehensive post-exacerbation follow up care that should focus on symptom control as well as preventive care to minimise future exacerbations [9]. Post-exacerbation care should involve smoking cessation support, medication and vaccination review, COPD action plans, assessment for long term oxygen therapy as well as referrals to required services including pulmonary rehabilitation (9). In Australia, post-exacerbation care of COPD patients is frequently managed by GPs. They are well suited to this role due to their ability to provide care continuity, coordination and integration with other health professionals and services. However, little evaluation of their care provision and uptake has been undertaken in the primary care settings where integration of evidence-based medicine and patient-centred care is intertwined.

This study therefore aimed to identify current general practice care for people soon after an acute exacerbation of COPD in Australia, examine GP satisfaction with communication from hospitals, determine familiarity with the COPD-X guidelines, and GPs knowledge and confidence in using these guidelines [9]. Finally, we aimed to identify barriers that GPs perceive limit the implementation of guidelines in post-discharge primary care for patients with COPD.

## Materials and methods

### Study design

A cross-sectional survey was disseminated electronically through multiple channels using Monash University’s secure Qualtrics survey platform. Participation in the study was voluntary with all participants provided with an explanatory statement describing the full nature of involvement. Informed implied consent was obtained from all individuals, denoted by their agreement to advance to commence the survey. All aspects of the study were reviewed and approved by the Human Research Ethics Committee of Monash University (MUHREC ref. 26571).

### Instrument design

Survey items were developed by the authors due to the absence of appropriate instruments to address the unique needs of this project. Content was based on the scientific literature and aligned to the COPD-X guidelines [9–13]. Questions explored current general practice care, barriers to implement evidence-based care (familiarity and confidence using guidelines, referrals to required services, ability of patients to see their GP within a timely manner), experiences of care following acute management in hospital as well as perspectives on potential improvements to optimise care in general practice. The survey consisted of both open and close ended questions. A respiratory physician with research experience examined the questionnaire for face validity. The survey was pilot tested with five GPs and questions were revised based on their feedback. A full copy of the survey is provided in the (S1 File), which details the items used to assess each domain of care described above.

### Participants & recruitment

Practising GPs in Australia who provided care for patients following an acute ex-acerbation of COPD in the 12 months prior to completing the survey were eligible to participate. The survey was distributed nationwide to maximise representativeness and generalisability. Initially we disseminated the online survey to a random selection of 1000 GPs registered on the Australasian Medical Publishing Company database, stratified by state and setting (Modified Monash Model (MMM) classification) [14]. Following an inadequate response, the survey was promoted using advertisements in newsletters of Primary Health Network, local faculties of Royal Australian College of General Practitioners (RACGP) and respiratory societies, on social media, and emails to personal contacts. Respondents were offered the opportunity to enter a prize draw for a gift voucher on survey completion. Survey responses were collected anonymously (and separately to the prize draw).

### Data analysis

Data were analysed descriptively using Stata SE16 software using appropriate summary statistics according to data type and distribution. Responses from five-point Likert-type scales were dichotomised (e.g. extremely and very familiar vs. moderately, slightly and not familiar at all) to allow cross-tabulation against participant sub- groupings and comparisons between groups were made using Chi squared tests. Our ability to perform subgroup analyses was limited by low participant number. Modelling and multivariate statistics were not attempted. Alpha was set at 0.05 for all analyses.

## Results and discussion

### Results

#### Demographics

**Fig 1:**
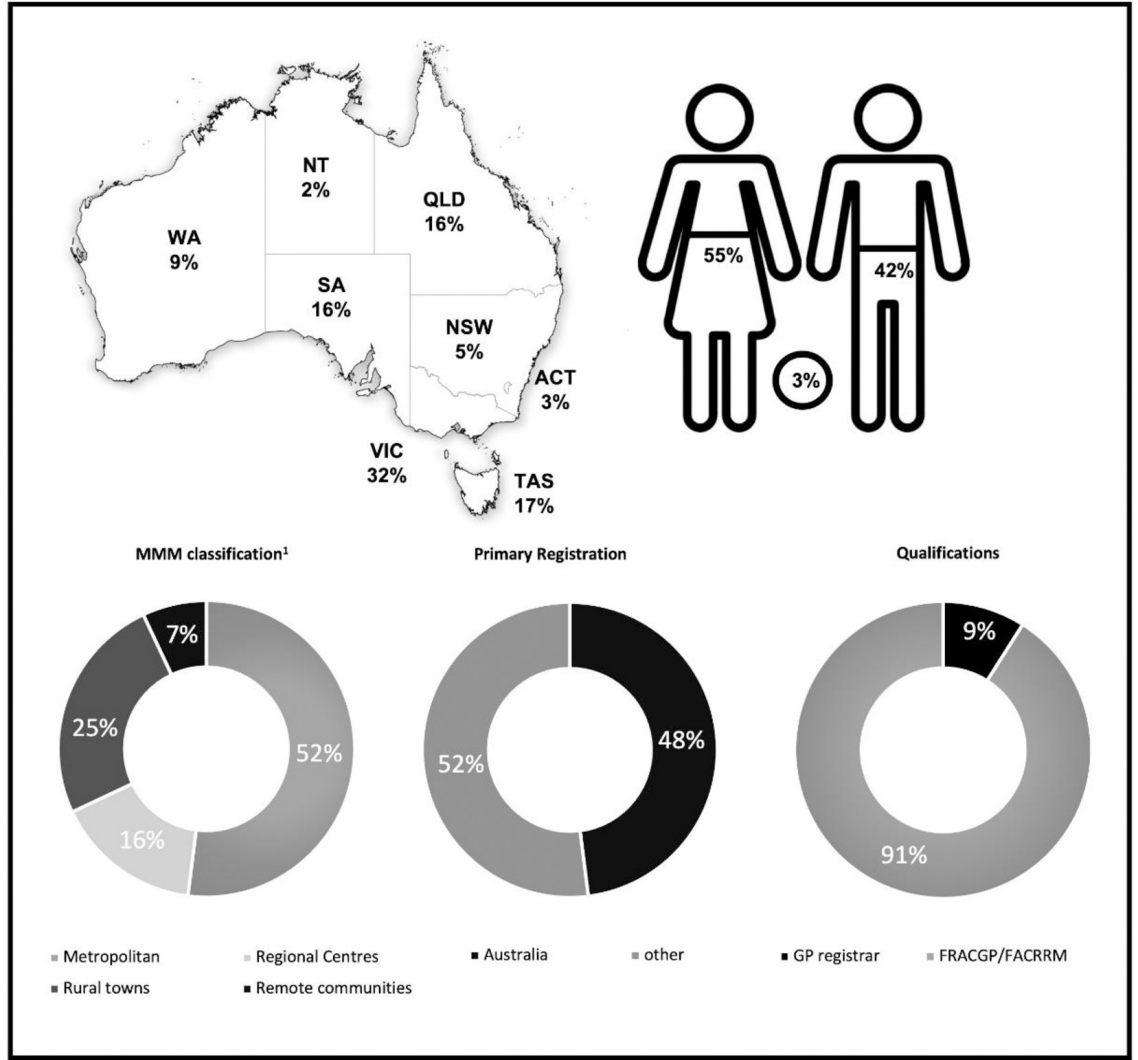
Respondents demographics.

Data were collected between 23 February 2021 and 14 October 2021. A total of 64 surveys were completed and received from all states and territories of Australia. The survey participants represented both Australian and international trained medical graduates as well as GP registrars and GPs with fellowship of RACGP (FRACGP) / Australian College of Rural and Remote Medicine (FACRRM) (Fig 1). Years of experience in general practice ranged from 3 to 47 years (Median IQR 14.5 (6–22.5)). The number of COPD patients with acute exacerbations seen in a typical 12-month period ranged from 3 to 101 (Median IQR 13 (11–31)).

More than 80% of surveyed GPs reported accessibility to most of the required services in their practice or community including pulmonary rehabilitation to manage COPD patients. However, allied health services such as dietitians, psychologists, occupational and speech therapists as well as pulmonary rehabilitation were less frequently used by GPs in managing COPD.

#### Current general practice care for people following an acute exacerbation of COPD

**Fig 2:**
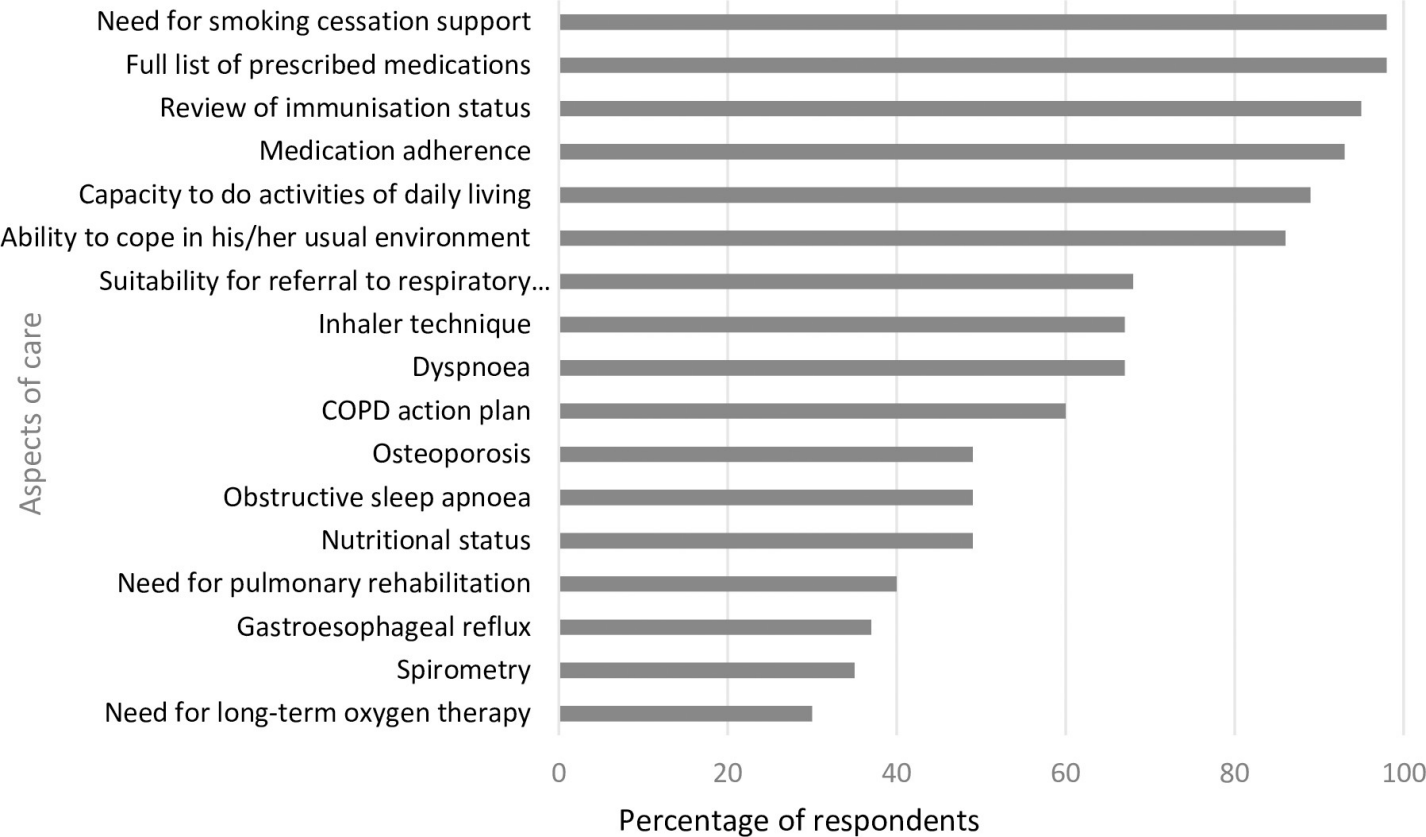
Aspects of care regularly assessed at GP visits following an acute exacerbation of COPD.

*Aspects of care regularly assessed at GP visits following an acute exacerbation of COPD*. More than 90% of GPs reported they regularly (‘always’ or ‘often’) assess the need for smoking cessation support, immunisation status, review the full list of prescribed medication at the follow up visit following an AECOPD. However, the survey revealed less than 50% of GPs consider other aspects of COPD care such as referral to pulmonary rehabilitation, spirometry, assessing long term oxygen therapy. Only 60% of respondents indicated that they initiated or reviewed COPD action plans at follow up visit (Fig 2)

**Fig 3:**
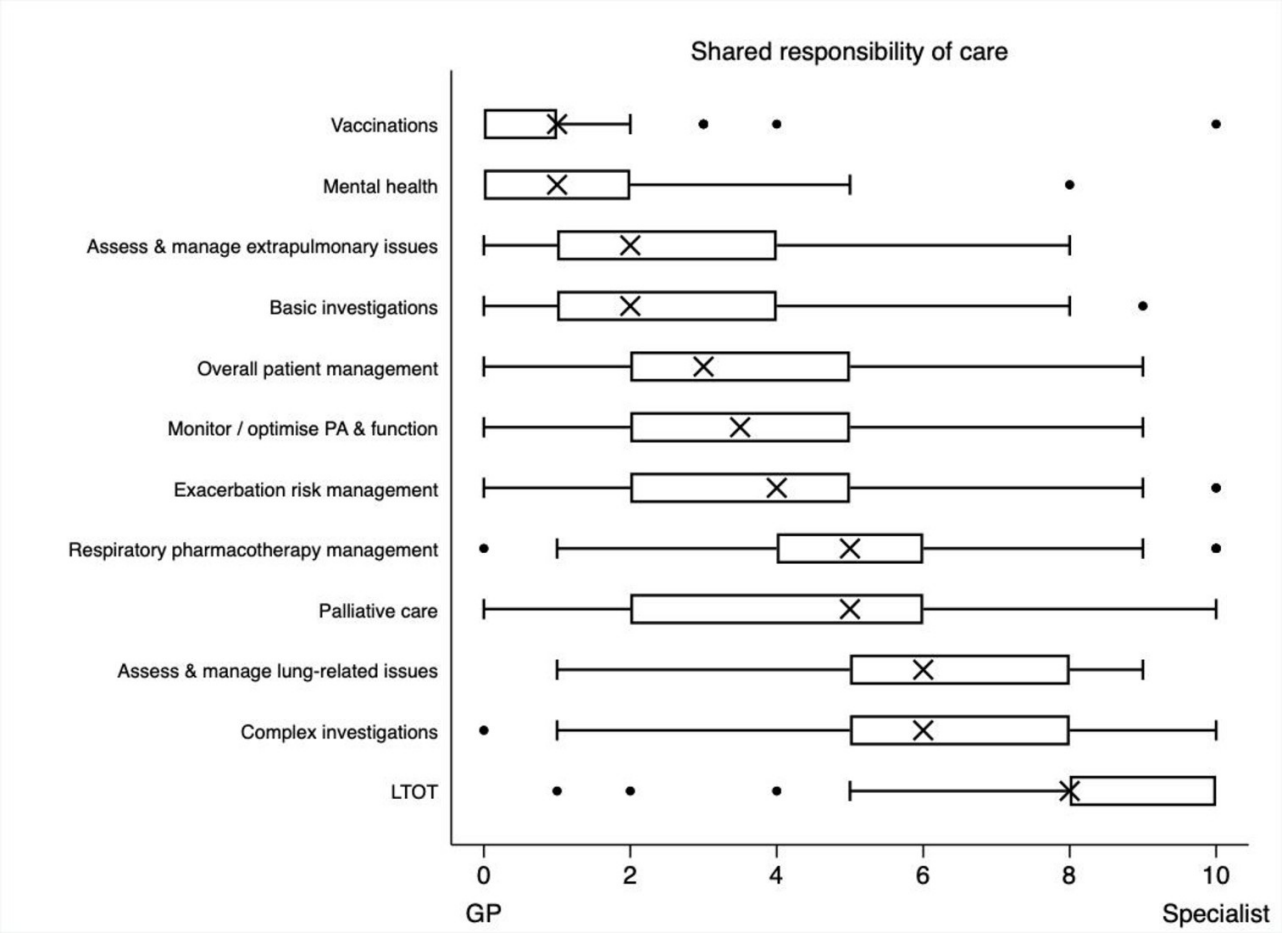
GPs perceptions of who should have responsibility for different aspects of care for COPD patients.

*Referral to respiratory specialists and GP perceived shared responsibility of care*. Most GPs (66%) envisaged respiratory specialists as having a key responsibility for caring for severe COPD patients but felt those with mild and moderate severity were best managed by GPs. Specific aspects of care of COPD perceived to be the primary responsibility of specialists included long-term oxygen therapy, performing complex investigations, assessing and managing other lung related issues (Fig 3).

#### Familiarity and confidence with COPD-X guidelines

**Fig 4:**
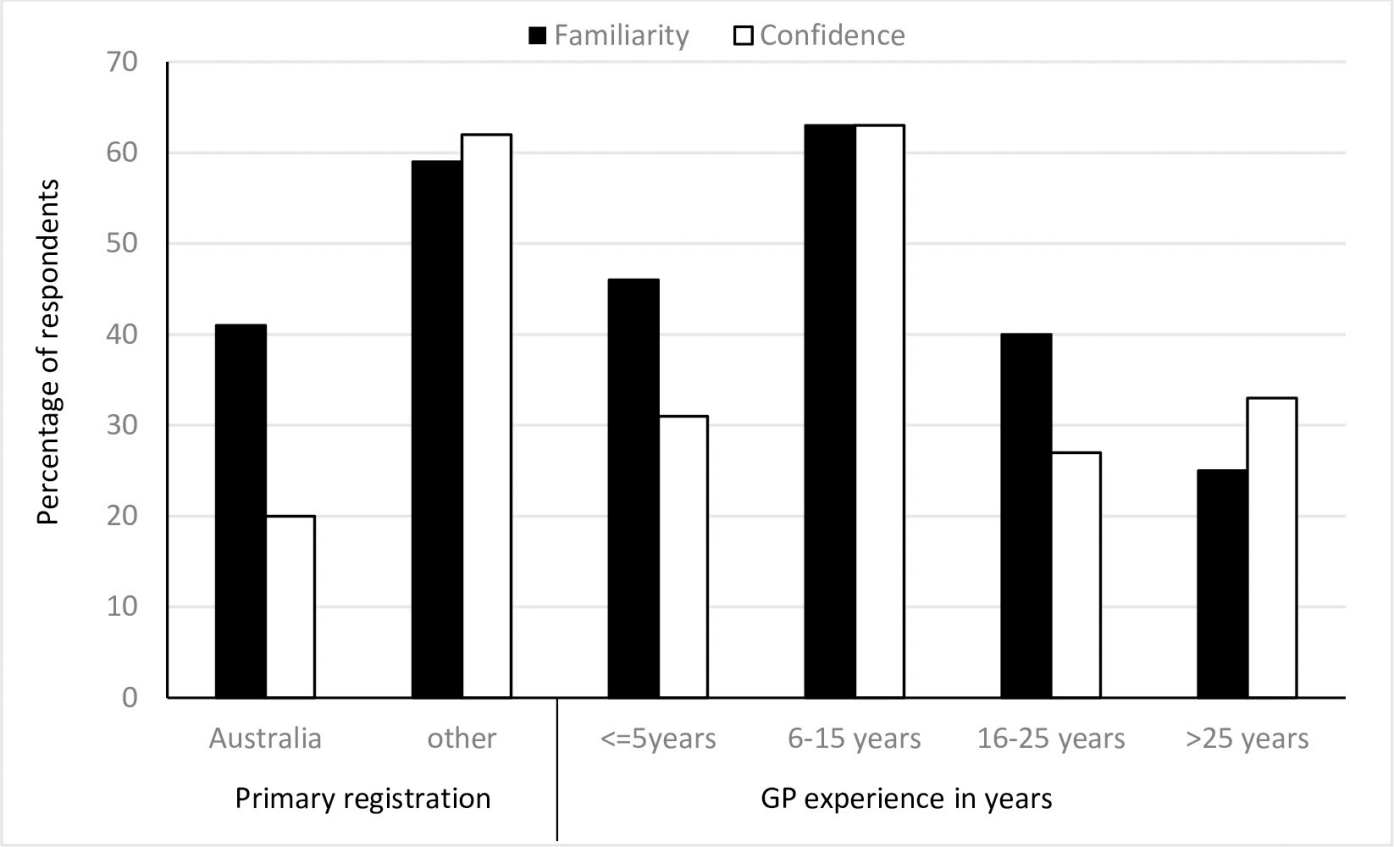
Familiarity with and confidence to use the COPD-X guidelines according to primary registration location and experience.

Less than half of surveyed GPs indicated they were extremely or very familiar with COPD-X guidelines and fewer still were confident in their implementation. The proportion of GPs who self-reported positive levels of familiarity with and confidence to use these guidelines was numerically higher in those who obtained their primary medical degree outside Australia and GPs with experience between 6–15 years compared to others. The only observation to reach statistical significance, however, was that pertaining to overseas-trained GPs with respect to confidence (p = 0.001) (Fig 4).

Surveyed GPs were asked whether they feel that any aspects of the COPD-X guidelines could be improved to enhance their usefulness in general practice as a free text response. While most participants (43%) felt no change was required, 12% were uncertain whether the guidelines needed to be changed and a small number (n = 4, 9%) commented they preferred to use ‘Therapeutic Guidelines’ (an independent resource of evidence-based treatment guidance for various medical conditions) rather than COPD-X [15]. Factors such as easy access during consultation (e.g., availability of a hard copy or more user-friendly website), and simplicity (e.g. algorithms, less words) were cited as potential strategies to improve implementation of guidelines into clinical practice by 36% of respondents.

#### Transitional care from hospital to community post-discharge care

**Fig 5:**
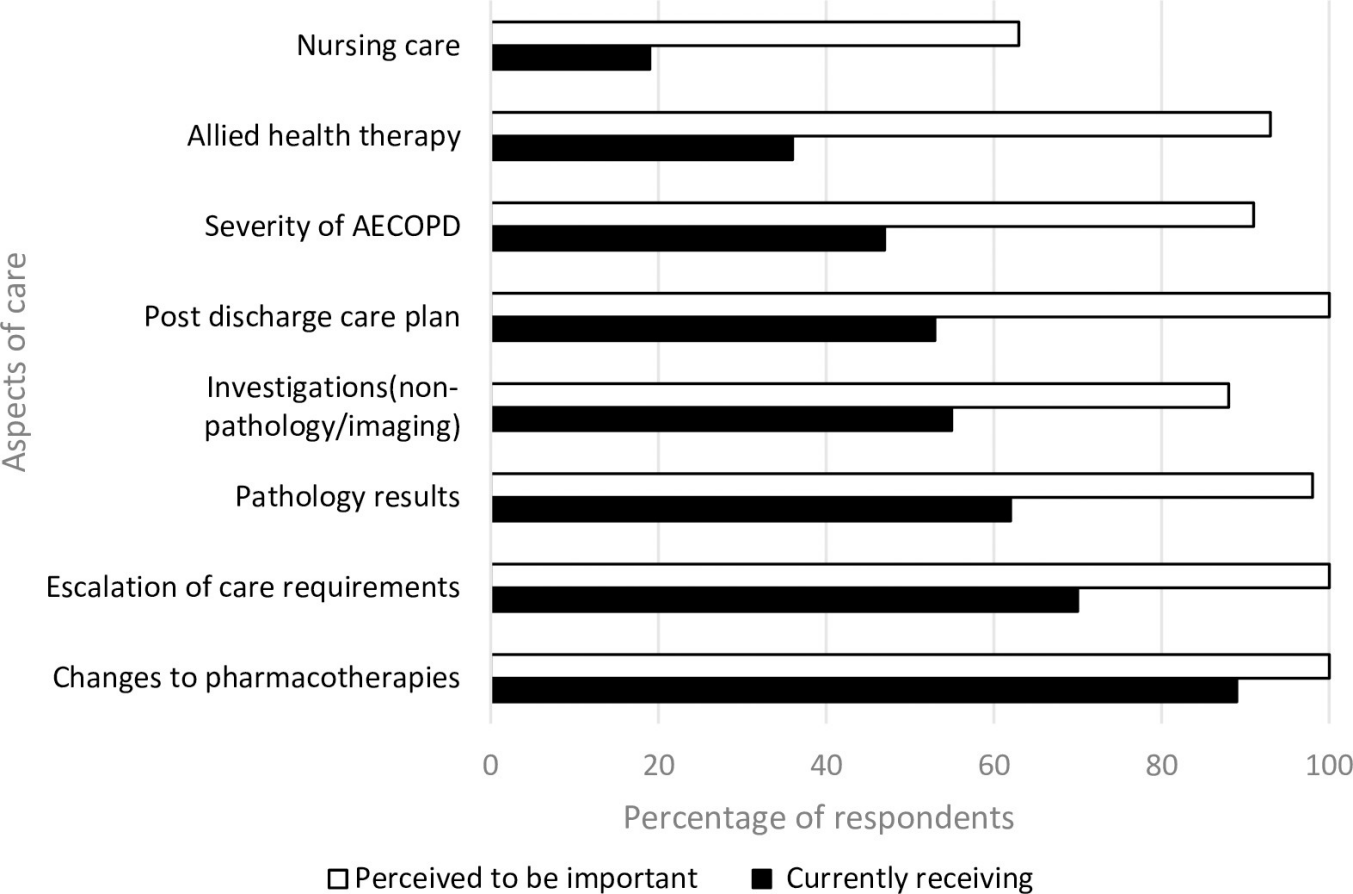
Content of discharge summaries perceived to be important compared to that vs what was currently provided in a typical discharge summary.

Surveyed GPs reported seeing approximately half of patients within 7 days of hospital discharge, The primary reason for this was a lack of awareness that their patients had experienced an exacerbation. Hospital discharge summaries were the key communication tool between hospital and GPs however only 47% of GP participants reported that they provided the information required to deliver effective post-discharge care. GPs reported several areas where content they perceived important were not adequately provided in discharge summaries such as information regarding allied health therapies, post-discharge planning and the severity of the exacerbation of AECOPD (Fig 5).

GPs who had provided care for hospitalised COPD patients were asked to provide their views on optimising transition care from hospital to community. More than half reported that they felt a clinical hand over would be useful. They valued direct verbal communication from an experienced medical practitioner from the hospital treating team. Others pointed out the importance of receiving timely discharge summaries with relevant and clear information on post discharge planning, and any referrals to specialists or allied health services made prior to discharge. Of concern, 77% of GPs reported feeling inadequately supported to deliver optimal care for patients following hospitalised exacerbations of COPD.

## Discussion

This study provides a contemporary account of GP care for patients following AECOPD in Australia. Findings suggest inconsistencies in care provision between GPs at follow up visits and a lack of familiarity with the principal Australian COPD guidelines. Discharge summaries remain the primary source of information regarding hospitalised care, however our sample of GPs reported these lacked critical information to help ensure the delivery of high-quality evidence-based care for these patients.

Our findings highlight the importance of supporting GPs to develop awareness of, and adhere to, evidence-based clinical practice guidelines. Variability of adherence and barriers to implement guidelines due to various reasons including unfamiliarity with recommendation, concerns of underlying evidence and applicability have been identified in prior research with little apparent improvement over time [16–18]. This is a concerning issue warranting further understanding and action and supported by previous Australian studies [19,20]. It is vital to identify (a) specific barriers to adhere/implement guidelines and (b) preferences for interventions to improve adherence among target users [17,21]. Continuous medical education opportunities that are perceived as acceptable and appealing to GPs may be one option to address such issues. Previous general practice research shows interactive small group educational meetings as a preferred intervention [21]. Audits and feedback have also been identified as effective methods to understand guidelines and identify personal performance gaps [21,22].

Our data suggest GPs more readily manage some aspects of patient care (e.g., vaccination, medication review) than others (e.g., spirometry, pulmonary rehabilitation) (Fig 1). Less familiarity and adherence to evidence-based guidelines and practical issues such as access to resources, familiarity with referral process may contribute to such practice variation [13,23]. As comprehensive management is essential following exacerbation of COPD to prevent future exacerbations and hospital admissions, such variability is important to address (9). Improvements to the shared coordination of care between hospitals, GPs, respiratory specialists and other community health services therefore remain an important focus of future research in this field. A review of clinical handover practices might be a sensible place to start. Although electronic communication systems improve the timely transfer of information from hospital to primary care setting, GPs still struggle to access clinically important information [24]. Development of tools such as standardised AECOPD-specific discharge summary templates could potentially help address this issue at the point of hospital discharge. Arranging a follow up appointment with patient’s nominated GP should be included in discharge planning as per COPD-X guidelines [9]. The lack of GPs’ awareness of hospital admissions, however, emphasises the need for communication to commence earlier during admissions and via additional methods. Verbal handovers are one suggestion that warrants further consideration, with international data suggesting this approach may be feasible [25].

### Strengths and limitations

A major issue that affected our sample size was the unavoidable conflict of survey timing with respect to Australia’s COVID-19 pandemic responses, with competing time demands related to the GP-led national vaccination rollout frequently communicated as a barrier to completion. Despite this, responses were achieved from all Australian States and Territories. The demographic characteristics of the participants (i.e. gender, primary medical registration status) in the study was comparable to the general population of GPs with only minor differences [26,27]. Our inclusion of snowballing methods of recruitment meant determining an accurate response rate was not possible. While the low participant number limits our ability to generate robust conclusions and perform sub-group analyses, we feel the insights learned from this study offer valuable perspectives of GP management for people following AECOPD. Our analyses were subsequently limited to exploratory and descriptive statistics. We did not use imputation for missing data as it added little value. It would be useful to repeat this survey in the future when COVID is not such a dominating issue. Finally, in this study, we have not measured the factors affecting accessibility to other health services, which may also have an impact on evidence-based care.

## Conclusions

Data from this survey of GP perceptions suggest there may be a role for supporting GPs to increase awareness or familiarity with COPD guidelines to inform their clinical practice. There appears an ongoing need to optimise GPs’ familiarity with dis-ease-specific guidelines, potentially via continuous professional development activities. Improvements to the clinical handover process from hospital to primary care remain an urgent issue to address, in order to help GPs, improve care during the critical post-hospitalisation period for people with AECOPD.

## Supporting information

S1 File A full copy of the survey. (DOCX)
