# Supplementary material for: General practice care following acute exacerbations of COPD: A survey of Australian general practitioners
Source: PLoS One. 2023 Apr 25;18(4):e0284731. doi: 10.1371/journal.pone.0284731 (PMC10129000; doi:10.1371/journal.pone.0284731)
Supplement: S1 File — (DOCX) [file pone.0284731.s001.docx]

S1 file: A full copy of the survey

General practice management of patients following acute exacerbations of COPD

Start of Block: Introduction and eligibility

Questions in

|  | Current general practice care |
| --- | --- |
|  | Demographics |
|  | Familiarity and adherence to guidelines |
|  | Transition of care from hospital to community |

the survey has been colour coded to indicate which objectives or areas of practice they were used to assess.

| Page Break |  |
| --- | --- |

Q1.2 During the past 12 months, have you been responsible for the management of COPD patients who experienced an acute exacerbation and required admission to hospital for their clinical management?

- Yes (1)
- No (2)

Skip To: Q1.3 If Q1.2 = 2

Skip To: Q1.5 If Q1.2 = 1

Q1.3 During the past 12 months, have you been responsible for the management of patients who experienced an acute exacerbation and were managed solely in a community-based setting (i.e. did not require hospitalisation)?

- Yes (1)
- No (2)

Skip To: Q1.5 If Q1.3 = 1

Skip To: Q1.4 If Q1.3 = 2

Q1.4 Thank you for responding to the eligibility questions. As you have responded ‘No’ to the above questions, unfortunately you are not eligible to take part in the survey. We very much appreciate your time and your interest in this research. You may now close the survey.

Skip To: End of Survey If Q1.4 Is Displayed

Q1.5 Based on your responses, you are eligible to participate. Please proceed to complete the survey.

End of Block: Introduction and eligibility

Start of Block: About you and your practice

Q2.1 **About you and your practice.**

Q2.2 What is the location of your practice?

|  |
| --- |

- ACT (1)
- NSW (2)
- NT (3)
- QLD (4)
- SA (5)
- TAS (6)
- VIC (7)
- WA (8)

Q2.3 Postcode

|  |
| --- |

________________________________________________________________

Q2.4 With which gender do you identify?

|  |
| --- |

- Male (4)
- Female (5)
- Non-binary (6)
- Prefer not to say (7)

| 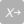 |
| --- |

Q2.5 From which country did you obtain your primary medical degree?

|  |
| --- |

▼ Australia (1) ... Zimbabwe (1357)

Q2.6 What qualification do you possess for practising in general practice in Australia (select all that apply)?

|  |
| --- |

- International Medical Graduate (IMG), no GP fellowship (1)
- GP registrar (2)
- FRACGP (3)
- FACRRM (4)

Q2.7 How many years have you been working as a GP?

|  |
| --- |

▼ <1 (1) ... >50 (52)

Q2.8 Approximately how many patients do you see for the management of acute exacerbations of COPD in a typical year (Note: the COVID-affected 2020 year may not have been a 'typical' year)?

|  |
| --- |

▼ 1 (1) ... >200 (202)

Q2.9 Apart from the provision of standard GP/patient consultations at your practice, what other services do you provide for patients that may be relevant for people with acute exacerbation of COPD (select all that apply)?

|  |
| --- |

- No other services (6)
- Home visits (1)
- Residential aged care facility visits (2)
- Hospital care (3)
- After hours (4)
- Telehealth (5)
- Other (please state) (7) ________________________________________________

Q2.10 Tell us about services available to you to manage people who have COPD.

|  |
| --- |

*Please note you may need to scroll to the right if viewing this question on a mobile device*

|  | Available in my practice | | Accessible in my community | | How often do you utilise this service? | | | | |
| --- | --- | --- | --- | --- | --- | --- | --- | --- | --- |
|  | Yes (1) | No (2) | Yes (1) | No (2) | Always (1) | Often (2) | Sometimes (3) | Rarely (4) | Never (5) |
| Lung function testing (1) |  |  |  |  |  |  |  |  |  |
| Arterial blood gases (2) |  |  |  |  |  |  |  |  |  |
| Practice nurse (3) |  |  |  |  |  |  |  |  |  |
| Exercise specialist (e.g. physiotherapist, exercise physiologist) (4) |  |  |  |  |  |  |  |  |  |
| Dietitian (5) |  |  |  |  |  |  |  |  |  |
| Psychologist (6) |  |  |  |  |  |  |  |  |  |
| Social worker (15) |  |  |  |  |  |  |  |  |  |
| Occupational therapy (12) |  |  |  |  |  |  |  |  |  |
| Speech therapy (19) |  |  |  |  |  |  |  |  |  |
| Imaging / radiology (14) |  |  |  |  |  |  |  |  |  |
| Immunisation clinic (7) |  |  |  |  |  |  |  |  |  |
| Smoking cessation support (8) |  |  |  |  |  |  |  |  |  |
| Pulmonary rehabilitation programs (16) |  |  |  |  |  |  |  |  |  |
| Chronic disease/case management programs (9) |  |  |  |  |  |  |  |  |  |
| Patient/peer support groups (10) |  |  |  |  |  |  |  |  |  |
| Aboriginal and Torres Strait Islander liaison (17) |  |  |  |  |  |  |  |  |  |
| Communication support for people with culturally and linguistically diverse backgrounds (18) |  |  |  |  |  |  |  |  |  |
| Other (11) |  |  |  |  |  |  |  |  |  |

End of Block: About you and your practice

Start of Block: About COPD and acute exacerbations

Q3.1 **About COPD management and acute exacerbations.**

 Do you have access to respiratory physician care for patients with COPD in your community/region?

|  |
| --- |

- Yes (1)
- No (2)

Display This Question:

If Q3.1 = 1

Q3.2
How often do you refer COPD patients with a current or previous history of acute exacerbations (whether managed in the community or in hospital) to a respiratory physician?

|  |
| --- |

|  | Always (1) | Often (2) | Sometimes (3) | Rarely (4) | Never (5) |
| --- | --- | --- | --- | --- | --- |
| Mild COPD (1) |  |  |  |  |  |
| Moderate COPD (2) |  |  |  |  |  |
| Severe COPD (3) |  |  |  |  |  |

Display This Question:

If Q3.1 = 1

Q3.3 For patients with COPD whom you feel are appropriate for referral to respiratory physicians, please describe your perceptions regarding the shared responsibility of care for the following aspects of care:

|  |
| --- |

|  | General practitioner's responsibility | Equally shared responsibility | Respiratory specialist responsibility |
| --- | --- | --- | --- |

|  | 0 | 1 | 2 | 3 | 4 | 5 | 6 | 7 | 8 | 9 | 10 |
| --- | --- | --- | --- | --- | --- | --- | --- | --- | --- | --- | --- |

| Overall patient management () | 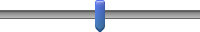 |
| --- | --- |
| Assessment and management of lung-related issues () | 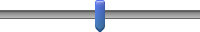 |
| Assessment an management of 'extrapulmonary' issues (not directly related to the lungs) () | 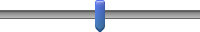 |
| Vaccinations () | 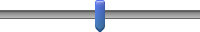 |
| Exacerbation risk management () | 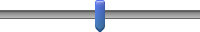 |
| Monitoring / optimising physical activity and function () | 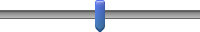 |
| Mental health () | 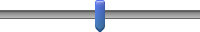 |
| Basic investigations (e.g. simple imaging, pathology) () | 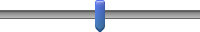 |
| Complex investigations (e.g. CT, echo) () | 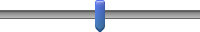 |
| Respiratory pharmacotherapy management (e.g. inhalers, steroids) () | 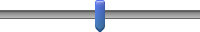 |
| Long term oxygen therapy () | 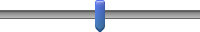 |
| Palliative care () | 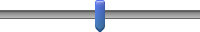 |

Q3.4 How familiar are you with Australia and New Zealand’s COPD-X guidelines for the management of COPD?

|  |
| --- |

- Extremely familiar (1)
- Very familiar (2)
- Moderately familiar (3)
- Slightly familiar (4)
- Not familiar at all (5)

Q3.5 How confident do you feel in your ability to implement COPD-X Guidelines in your general practice for patients with acute exacerbations of COPD (whether managed in the community or in hospital)?

|  |
| --- |

- Extremely confident (1)
- Very confident (2)
- Moderately confident (3)
- Slightly confident (4)
- Not confident at all (5)

Q3.6 Please tell us whether you feel any aspects of the COPD-X guidelines could be improved to enhance their usefulness for the **general practitioner** management of patients with acute exacerbations of COPD (whether managed in the community or in hospital):

|  |
| --- |

________________________________________________________________

Q3.7 For COPD patients who have experienced a recent acute exacerbation (whether managed in the community or in hospital), please indicate how often you assess the following aspects of care in your general practice:

|  |  |
| --- | --- |

|  | Always (1) | Often (2) | Sometimes (3) | Rarely (4) | Never (5) |
| --- | --- | --- | --- | --- | --- |
| Ability to cope in his/her usual environment (1) |  |  |  |  |  |
| Capacity to do activities of daily living (2) |  |  |  |  |  |
| Dyspnoea (e.g. Modified Medical Research Council Dyspnea Scale) (3) |  |  |  |  |  |
| Nutritional status (4) |  |  |  |  |  |
| Spirometry (e.g. FEV1%) (5) |  |  |  |  |  |
| Full list of prescribed medications (6) |  |  |  |  |  |
| Medication adherence (7) |  |  |  |  |  |
| Inhaler technique (8) |  |  |  |  |  |
| Need for long-term oxygen therapy (9) |  |  |  |  |  |
| Need for pulmonary rehabilitation (10) |  |  |  |  |  |
| Suitability for referral to respiratory physician (11) |  |  |  |  |  |
| Gastroesophageal reflux (12) |  |  |  |  |  |
| Obstructive sleep apnoea (13) |  |  |  |  |  |
| Osteoporosis (14) |  |  |  |  |  |
| Need for smoking cessation support (15) |  |  |  |  |  |
| Review of immunisation status (influenza and pneumococcal) (16) |  |  |  |  |  |
| COPD action plan (17) |  |  |  |  |  |

End of Block: About COPD and acute exacerbations

Start of Block: About patients with recent hospitalisation due to acute exacerbation of COPD(AEC

Display This Question:

If Q1.2 = 1

Q4.1 **About acute exacerbations of COPD that require hospitalisation.**

 How are you typically alerted if one of your patients with COPD is **hospitalised** for an acute exacerbation (select all that apply)?

|  |
| --- |

- Notification upon admission/electronic alert from hospital system (1)
- Notification on discharge/ electronic alert from hospital system (2)
- Phone calls during admission (e.g. medical team enquiries) (3)
- When a patient requesting an appointment (4)
- No systematic method (5)
- I am usually the person who admits them to my local hospital (6)
- Other (please state) (________________________________________________

Display This Question:

If Q1.2 = 1

Q4.2 How do you receive correspondence regarding details of an acute exacerbation of COPD for patients **admitted to hospital** (e.g. discharge summaries)?

|  |
| --- |

- Phone call (6)
- Fax (2)
- E-mail (3)
- Secure electronic health communication system (non-email) (4)
- Paper letter sent via post (1)
- Paper letter provided directly via patient (8)
- I am not typically informed about hospitalisations for my patients (5)
- I am usually the person who managed them in my local hospital (9)
- Other (please state) (7) 2________________________________________________

Display This Question:

If Q1.2 = 1

Q4.3 Approximately how many days after hospital discharge do you typically receive discharge summaries?

|  |
| --- |

▼ <1 (1) ... >30 (32) 3

Display This Question:

If Q1.2 = 1

Q4.4 Approximately how often is receipt of a discharge summary from hospital the first alert that one of your COPD patients has experienced an acute exacerbation?

|  |
| --- |

- 80-100% of the time (1)
- 60-79% of the time (2)
- 40-59% of the time (3)
- 20-39% of the time (4)
- 0-19% of the time (5)

Display This Question:

If Q1.2 = 1

Q4.5 How are follow-up GP consultations typically coordinated following hospital discharge for an acute exacerbation of COPD (select all that apply)?

|  |
| --- |

- Hospital contact the practice to book appointments (1)
- Practice calls the patient to book an appointment (2)
- Patient contacts practice to book an appointment (3)
- Other (Please specify) ____(4)___________________________________________

Display This Question:

If Q1.2 = 1

Q4.6 In general, for patients who present to your general practice following hospitalisation due to acute exacerbation of COPD, what kind of consultation do you feel is most appropriate?

|  |
| --- |

- Standard consultation (1)
- Long consultation (2)
- Chronic disease management consultation (3)
- Other (4) ________________________________________________

Display This Question:

If Q1.2 = 1

Q4.7 Approximately how many days after discharge from hospital do you typically see COPD patients following an acute exacerbation?

|  |  |  |
| --- | --- | --- |

▼ 1 (1) ... >30 (31) 5

Display This Question:

If Q1.2 = 1

Q4.8 Of your COPD patients who experience an acute exacerbation, requiring hospitalisation, approximately what proportion do you estimate you see within 7 days of discharge from hospital?

|  |
| --- |

- 80-100% (1)
- 60-79% (2)
- 40-59% (3)
- 20-39% (4)
- 0-19% (5)

Display This Question:

If Q1.2 = 1

Q4.9 What are the typical reasons for scheduling appointments for patients following discharge from hospital due to an exacerbation of COPD (select all that apply)?

|  |
| --- |

- Instructed to do so from hospital (e.g. follow-up care, test results, imaging, blood monitoring post steroids) (1)
- Review/advice regarding prescribed medications (related to their acute exacerbation of COPD) (2)
- Review/advice regarding prescribed medications (unrelated to their acute exacerbation of COPD) (3)
- Uncertainties regarding their management (4)
- Need for additional services/care (5)
- To discuss ongoing other concerns related to their recent acute exacerbation of COPD (6)
- New issues developed since discharge (7)
- Patients wishing to 'keep you in the loop' (8)
- Other (Please specify) (9) 2________________________________________________

Display This Question:

If Q1.2 = 1

Q4.10 In COPD patients you see following discharge from hospital due to an acute exacerbation, how often do you access hospital discharge summaries?

|  |
| --- |

- Always (1)
- Often (2)
- Sometimes (3)
- Rarely (4)
- Never (5)

Display This Question:

If Q1.2 = 1

Q4.11 How useful do you find hospital discharge summaries for providing adequate information to inform your follow-up general practice management?

|  |
| --- |

- Extremely useful (1)
- Very useful (2)
- Moderately useful (3)
- Slightly useful (4)
- Not at all useful (5)

Display This Question:

If Q1.2 = 1

Q4.12 Regarding hospital discharge summaries:

|  |
| --- |

*Please note you may need to scroll to the right if viewing this question on a mobile device*

|  | How often are the following aspects of care described in text-based discharge summaries? | | | | | Do you think this is an important item affecting your management following discharge | |
| --- | --- | --- | --- | --- | --- | --- | --- |
|  | Always (1) | Often (2) | Sometimes (3) | Rarely (4) | Never (5) | Yes (1) | No (2) |
| Severity of acute exacerbation of COPD (1) |  |  |  |  |  |  |  |
| Escalation of care requirements (e.g. mechanical / non-invasive ventilation) (2) |  |  |  |  |  |  |  |
| Changes to pharmacotherapies (3) |  |  |  |  |  |  |  |
| Pathology results (5) |  |  |  |  |  |  |  |
| Investigations (non-pathology/imaging) (4) |  |  |  |  |  |  |  |
| Relevant aspects of allied health therapy (6) |  |  |  |  |  |  |  |
| Relevant aspects of nursing care (7) |  |  |  |  |  |  |  |
| Post discharge care plan (8) |  |  |  |  |  |  |  |

Display This Question:

If Q1.2 = 1

Q4.13 For your COPD patients who require hospitalisation due to their acute exacerbations, what factors commonly contribute to them **NOT** seeking GP follow-up with you after discharge (select all that apply)?

|  |
| --- |

- GP unaware of hospitalisation (1)
- Patient never referred (2)
- Patient referred but did not attend scheduled appointment (3)
- Timely appointment not available (4)
- Patient recovered (5)
- Patient referred to respiratory specialist (6)
- Patient too unwell to come to the practice (7)
- Patient attended to a different GP (8)
- Reason(s) unclear (9)
- Other (please specify) (10) 3________________________________________________

Display This Question:

If Q1.2 = 1

Q4.14
Overall, how supported do you feel as a general practitioner to deliver optimal care of patients following hospitalised exacerbations of COPD?

|  |
| --- |

- Extremely supported (1)
- Very supported (2)
- Moderately supported (3)
- Slightly supported (4)
- Not supported (5) 4

End of Block: About patients with recent hospitalisation due to acute exacerbation of COPD(AEC

Start of Block: Barriers/facilitators to care after an acute exacerbation of COPD

Display This Question:

If Q1.2 = 1

Q5.1 What improvements would you suggest to optimise the transition of care from hospital to your general practice following hospitalisation due to an acute exacerbation of COPD?

|  |
| --- |

________________________________________________________________

Improved discharge summaries

Display This Question:

If Q1.2 = 1

Q5.2 How useful would you find a verbal handover (e.g. phone) from a member of the hospital-based medical team to discuss relevant details regarding COPD patients’ admission due to an acute exacerbation?

|  |
| --- |

- Extremely useful (1)
- Very useful (2)
- Moderately useful (3)
- Slightly useful (4)
- Not at all useful (5)

Display This Question:

If Q5.2 = 1

Or Q5.2 = 2

Or Q5.2 = 3

Q5.3 If you could access a verbal handover with a member of the medical team, WHO would you feel is the most appropriate medical team member to do this?

|  |
| --- |

- Respiratory physician (1)
- Respiratory registrar (2)
- Resident (HMO) (3)
- Medical student (4)
- Other (please state) (5) ___________________________________________

Display This Question:

If Q5.2 = 1

Or Q5.2 = 2

Or Q5.2 = 3

Q5.4 If you could access a verbal handover with a member of the medical team, WHEN would you feel is the most appropriate time for this to be completed (select all that apply)?

|  |
| --- |

- Upon admission to hospital (1)
- Early during the inpatient stay (2)
- Later during the inpatient stay, but prior to discharge (5)
- Upon discharge (3)
- In the first 48 hours post-discharge (4)
- Other (please state) (6) 1________________________________________________

End of Block: Barriers/facilitators to care after an acute exacerbation of COPD

Start of Block: End of survey

Q6.1 **Final survey details.**

 Thank you for your assistance to complete this survey. 
 
**Would you like to enter the prize draw to win a gift voucher of $250?**

- Yes (4)
- No (5)

Q6.2 **Would you be interested in participating in a follow-up qualitative interview to further explore GPs experiences and perspectives regarding the management of patients following AECOPDs?**


This would involve an interview with a member of the research team and is anticipated to last between 30 and 45 minutes, conducted by phone or video conference at a time of your convenience. Participants in this part of the project will be reimbursed with a $250 gift voucher for their time.

- Yes (4)
- No (5)

Display This Question:

If Q6.1 = 4

Or Q6.2 = 4

Q6.3 Thank you for your response(s). In order to obtain your personal details but ensure your responses from this survey remain anonymous, please click the following link to directed to a short separate Qualtrics web form: [https://monash.az1.qualtrics.com/jfe/form/SV_6D677LI71rNlaoR](about:blank)
 
This will result in completion of the survey.

End of Block: End of survey

https://www1.racgp.org.au/ajgp/2022/april/giving-asthma-support-to-patients-program-evaluation

https://www1.racgp.org.au/ajgp/2022/april/giving-asthma-support-to-patients-program-evaluati
